# Matrix Metalloproteinases System and Types of Fibrosis in Rat Heart during Late Pregnancy and Postpartum

**DOI:** 10.3390/medicina55050199

**Published:** 2019-05-23

**Authors:** Adolfo Virgen-Ortiz, Saraí Limón-Miranda, Diana Guadalupe Salazar-Enríquez, Valery Melnikov, Enrique Alejandro Sánchez-Pastor, Elena Margarita Castro-Rodríguez

**Affiliations:** 1 Universitary Center for Biomedical Research, University of Colima, C.P. 28045 Colima, Mexico; qbdiana_se@hotmail.com (D.G.S.-E.); espastor@ucol.mx (E.A.S.-P.); ecastro@ucol.mx (E.M.C.-R.); 2 Department of Chemical-Biological Sciences and Agropecuary, University of Sonora, C.P. 85880 Sonora, Mexico; sarai_limonm@hotmail.com; 3 Faculty of Medicine, University of Colima, C.P. 28045 Colima, Mexico; melnik@ucol.mx

**Keywords:** physiological cardiac remodeling, metalloproteinases, pregnancy, extracellular matrix, fibrosis

## Abstract

*Background and objectives:* Cardiac remodeling in pregnancy and postpartum is poorly understood. The aim of this study was to evaluate changes in cardiac fibrosis (pericardial, perivascular, and interstitial), as well as the expression of matrix metalloproteinases (MMP-1, MMP-2, and MMP-9) and their inhibitors (Tissue inhibitors of metalloproteinases, TIMP-1 and TIMP-4) during late pregnancy and postpartum in rat left ventricle. *Materials and Methods:* Female Sprague–Dawley rats were used for this study. Rats were divided three groups: non-pregnant, late pregnancy, and postpartum. The heart was weighed and cardiac fibrosis was studied by conventional histological procedures. The expression and transcript level of target proteins were evaluated using immunoblot techniques and quantitative PCR. *Results:* The experiments showed an increase of perivascular, pericardial, and interstitial fibrosis in heart during pregnancy and its reversion in postpartum. Moreover, in late pregnancy, MMP-1, MMP-2, and MMP-9 metalloproteinases were downregulated and TIMP-1 and TIMP-4 were upregulated in left ventricle. *Conclusions:* Our data suggest that the metalloproteinases system is involved in the cardiac extracellular matrix remodeling during pregnancy and its reversion in postpartum, this improves the knowledge of the adaptive cardiac remodeling in response to a blood volume overload present during pregnancy.

## 1. Introduction

Pregnancy is a physiological state where the cardiac remodeling induced by volume overload is of great interest for study. During pregnancy the circulating blood volume increases [1,2] and this creates a higher demand for the cardiovascular system to carry out functional, structural, and metabolic adaptations. Cardiac remodeling involves cellular and molecular changes including eccentric cardiac hypertrophy [3,4] without cardiac hyperplasia [5], decrease in the myocardial stiffness [4], increase in the action potential duration associated to reduction in outward K+ transient current density [3], increase in the cardiac contractility and angiogenesis [6], and shifts in the metabolism that are modulated by the activation of transcription factors as HIF-1α (Hypoxia inducible factor 1-α) and PPARγ (peroxisome proliferator-activated receptor γ) [7] and recently, it has been proposed that an increase in Fgf21 (Fibroblast growth factor-21) may play a key role in cardiac remodeling during pregnancy [8,9].

Physiological cardiac remodeling during the pregnancy and its reversion in postpartum has been little studied. The extracellular matrix is a structural complex fundamental for the cardiac activity providing a support to cardiomyocytes, and functionally has a regulatory role in the active tension generation and cardiac stiffness, these mechanical properties are crucial for heart performance during diastole [10]. In late pregnancy, reduction in the cardiac ventricular stiffness and reversion in postpartum has been reported [4], and this adaptive change contributes to improve the ventricular filling during diastole acting as a compensatory mechanism that allows handling a higher blood volume. The molecular mechanism involved in the cardiac stiffness regulation during the pregnancy is little understood. A recent study suggests that the decrease in cardiac stiffness during late pregnancy is due to a differential expression of collagen isoforms in left ventricle being type III collagen upregulated and type I collagen downregulated [11]. The synthesis and degradation of the extracellular matrix components are regulated by the metalloproteinases system and its inhibitors; however, there are very few studies that evaluate the role of the metalloproteinases system in pregnancy-induced cardiac remodeling.

In the present study, we evaluated fibrosis (pericardial, perivascular and interstitial), as well as the expression of matrix metalloproteinases (MMP-1, MMP-2, and MMP-9) and their inhibitors (TIMP-1 and TIMP-4) during late pregnancy and postpartum in rat left ventricle. Our data could help understand the molecular mechanisms involved in the pregnancy-induced cardiac extracellular matrix remodeling.

## 2. Materials and Methods

### 2.1. Animals

Animal care and experimental procedures were approved by the Bioethics Committee of the University of Colima (Approval number 2018-16), using guidelines based on the Guide for the Care and Use of Laboratory Animals (US Department of Health, NIH). Three-month-old female Sprague–Dawley rats were divided into three groups: controls or non-pregnant rats (NP, diestrus 258 ± 7.6 g, n = 10), late pregnant rats (LP, 21 days of gestation, 332 ± 10.9 g, n = 10), and postpartum rats (PP, 7 days, 270 ± 7.7 g, n = 10). All rats received free access to water and food and were maintained in acrylic cages on a 12:12-h light–dark cycle with a room temperature of 24 ± 1 °C and humidity of 60–70%. To obtain cardiac tissue samples, all rats were killed under anesthesia (pentobarbital sodium, 50 mg/kg, intraperitoneally) by cervical dislocation and then hearts were extirpated and weighed. Subsequently the left ventricle was dissected, immediately it was either fixed for histological analysis or frozen in liquid nitrogen and stored at −80 °C for posterior analysis. 

### 2.2. Histology

In order to investigate cardiac fibrosis, left ventricles were fixed in 10% buffered-formalin solution, embedded in paraffin, and processed for routine histological analysis. Tissue sections (5 μm) were stained using Masson’s trichrome protocol, and examined by optical microscopy using digital imaging with an Axioscope microscope, an MR5 digital camera, and Axiovision software version 4.1 (Carl Zeiss, Göttingen, Germany). Pericardial and perivascular fibrosis was quantified by measuring the thickness of the periphery (pericardial) and vessel surrounding layer (vascular) stained in blue, and posteriorly data were normalized with respect to the NP group. Interstitial fibrosis was measured as blue color intensity per tissue area and it expressed in percentage.

### 2.3. Matrix Metalloproteinase-1 (MMP-1) Measurement by ELISA 

MMP-1 level in total homogenates of left ventricular tissue in the three experimental groups (NP, LP, and PP) was measured using enzyme-linked immunosorbent assay (ELISA) kits, according to the manufacturer’s instructions (MMP-1, Biotrak Amersham Pharmacia Biotech, Buckinghamshire, United Kingdom). MMP-1 concentration in the extract was expressed as ng/mL/g total protein. Posteriorly, data were normalized with regard to NP group.

### 2.4. Tissue Inhibitor of Metalloproteinase-1 (TIMP-1) Transcript Levels by Quantitative Polymerase Chain Reaction (PCR)

Total RNA was isolated individually from frozen left ventricle samples using TRIzol reagent (Invitrogen) following the manufacturer’s instructions. RNA integrity was confirmed by measuring the absorbance at 260 nm/280 nm and by 1% agarose gel electrophoresis. Genomic DNA in total RNA was eliminated by digestion using recombinant DNase I (Roche, Indianapolis, IN, USA), as specified by the manufacturer. 100 ng of total RNA from each sample were submitted to reverse transcription and polymerase chain reaction (RT-PCR) using a Lightcycler RNA master SYBR Green I kit (Roche, Nutley, NJ, USA), specific primers for Timp-1 gene and GAPDH gene (reference gene), LightCycler capillaries and LightCycler 1.5 system (Roche, Nutley, NJ). Amplification reactions were performed to determine the efficiency of amplification for each primer pair. Timp-1 gene expression were normalized to glyceraldehyde 3-phosphate dehydrogenase (GAPDH) as a reference gene. Relative expression was calculated according to the equation 2^−ΔΔCt^, where ΔΔCt = ((Ct _Timp-1_ − Ct _GAPDH_) _problem tissue_ − (Ct _Timp-1_ − Ct _GAPDH_) _control tissue_)), and Ct is threshold cycle [12]. Analysis of the data was performed using LightCycler software. The following specific primers were used:

Tissue inhibitor of Metalloproteinase-1 (Timp-1) gene

F: 5′-CAGCGAGGAGTTTCTCATCG-3′
R: 5′-GGCTGAACAGGGAAACACTG-3′

Glyceraldehyde 3-phosphate dehydrogenase (GAPDH) gene

F: 5′-TCCCTCAAGATTGTCAGCAA-3′
R: 5′-GCAGTGATGGCATGGACT-3′

### 2.5. Inmunoblot Analysis

To evaluate protein expression levels, left ventricular tissue was homogenized in lysis buffer with the protease inhibitor cocktail included (Roche). Fifty micrograms of total proteins were separated by 10% SDS-PAGE. Separated proteins were electrotransferred to nitrocellulose membranes. Membranes were blocked with 5% blocking agent (GE healthcare, Amersham, Buckinghamshire, UK) and immunoblotted using the following antibodies: TIMP-1 (Catalog No. 21734 Santa Cruz Biotechnology), TIMP-4 (Catalog No. 30076 Santa Cruz Biotechnology), MMP-2 (Catalog No. 10736 Santa Cruz Biotechnology), MMP-9 (Catalog No. 6841R Santa Cruz Biotechnology), GAPDH was used as a load control (Catalog No. 25778 Santa Cruz Biotechnology). After that, secondary horseradish peroxidase-conjugated antibody was applied for 1 hour at room temperature. The blots were developed with a chemiluminescence detection system (GE Heathcare, Amersham, Buckinghamshire, UK) and visualized by exposure to Kodak radiographic film. Density of bands was measured with Image J (National Institutes of Health, Bethesda, MD, USA).

### 2.6. Statistical Analysis

All data were presented as means ± standard deviation. Normality test (Shapiro–Wilk) was applied and then the groups were compared using a one-way analysis of variance with post hoc tests of Bonferroni. The significance level used in the study was 95% (*p* ≤ 0.05). The data analysis was carried out with Sigmaplot software (version 10.0).

## 3. Results

### 3.1. Cardiac Hypertrophy and Fibrosis

During late pregnancy, the heart mass significant increased 30% compared with non-pregnant group, while in the postpartum it decreased respect to late pregnant group (*p* < 0.05) (Heart weight: NP, 0.85 ± 0.02 g; LP, 1.11 ± 0.03 g; PP, 0.93 ± 0.01 g). 

**Figure 1:**
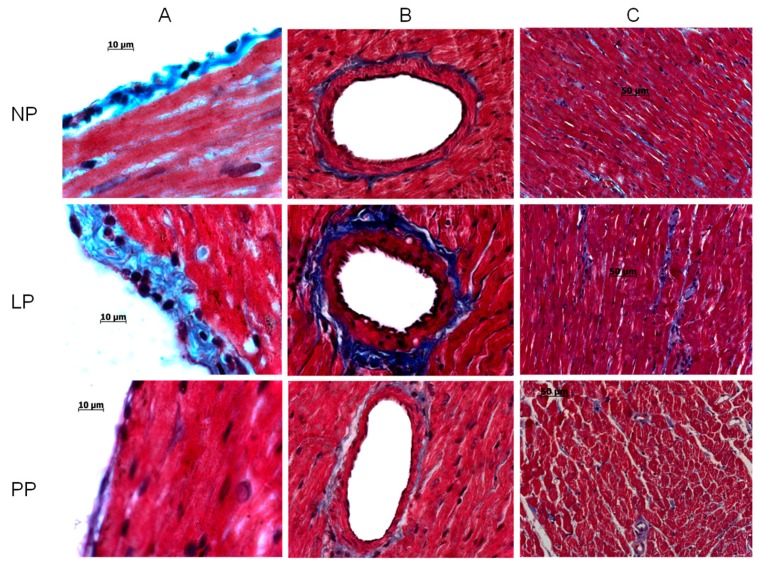
Heart histological illustrative sections stained with Masson’s trichrome to detect fibrosis (healthy myocardium, red; fibrotic tissue, blue). (**A**) Pericardial zone, (**B**) Perivascular zone and (**C**) Interstitial zone in non-pregnant (NP), late-pregnant (LP, 21 days) and rat postpartum (PP, 7 days).

The histological study in cardiac left ventricle of rats revealed pericardial fibrosis increased 3.4 fold in LP group compared with NP group (*p* < 0.05) (NP, 1 ± 0.2; LP, 3.4 ± 0.3; PP, 1.2 ± 0.2 fold). The vascular fibrosis was increased 2.6 fold in LP group compared with NP group (*p* < 0.05) (NP, 1 ± 0.1; LP, 2.6 ± 0.2; PP, 1.1 ± 0.1 fold). Finally, interstitial fibrosis also was increased 1.7 fold in LP group compared with NP group (*p* < 0.05) (NP, 2.4 ± 0.1%; LP, 4 ± 0.3%; PP, 2.2 ± 0.2%). All fibrosis were reversed in postpartum (Figure 1).

### 3.2. Metalloproteinases and Tissue Inhibitor of Metalloproteinases (TIMPs)

**Figure 2:**
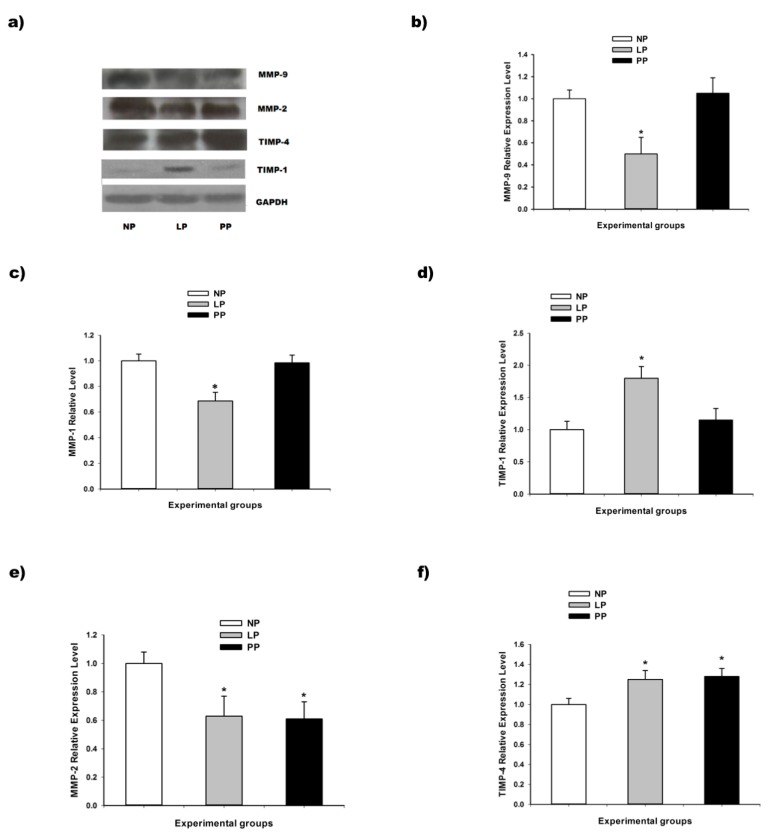
Protein expression of metalloproteinases and endogenous inhibitors. (**a**) Illustrative Western blots of glyceraldehyde 3-phosphate dehydrogenase (GAPDH) (37 kDa), tissue inhibitor of metalloproteinase-1 (TIMP-1) (23 kDa), TIMP-4 (26 kDa), matrix metalloproteinase-2 (MMP-2) (63 kDa), MMP-9 (92 kDa); (**b**) MMP-9 expression; (**c**) MMP-1 expression; (**d**) TIMP-1 expression; (**e**) MMP-2 expression; (**f**) TIMP-4 expression. Experimental groups: non-pregnant (NP), late-pregnant (LP, 21 days), and rat postpartum (PP, 7 days). The data presented a normal distribution (Shapiro–Wilk test), posteriorly One-Way ANOVA was used to compare the three groups, then comparison between pair were performed with Bonferroni test. * Significant difference with *p* < 0.05 respect to NP group.

The current study shows that MMP-1, MMP-2, and MMP-9 expression was lower in left ventricle of pregnant rats than in the non-pregnant group (Figure 2), while in the postpartum MMP-1 and MMP-9 expression was similar to NP group (Figure 2); MMP-2 expression was less in PP group compared with NP group (Figure 2). These experimental results confirmed the participation of metalloproteinases in the cardiac remodeling of the extracellular matrix in pregnancy and postpartum.

**Figure 3:**
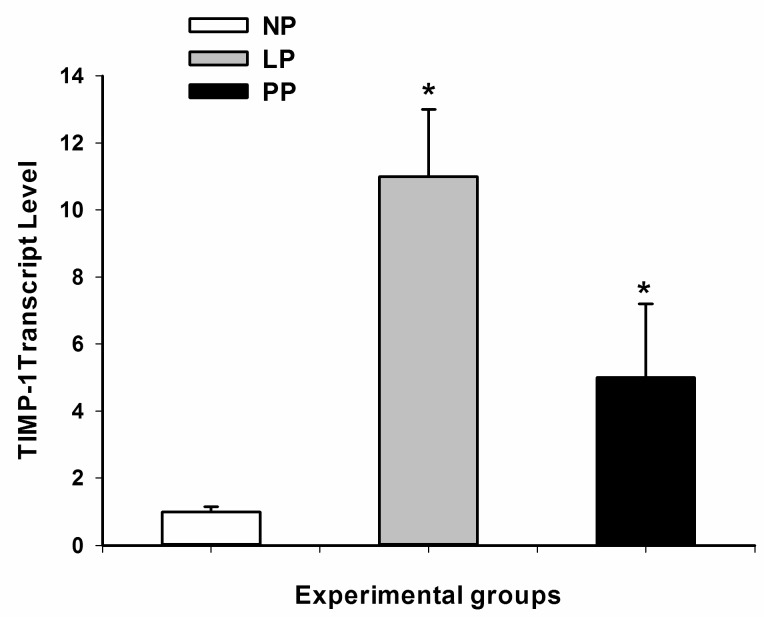
Tissue inhibited metalloproteinase-1 (TIMP-1) transcriptional level in left ventricle during pregnancy and postpartum. Non-pregnant (NP, n = 8), late-pregnant (LP, 21 days, n = 8), and rat postpartum (PP, 7 days, n = 8). The data presented a normal distribution (Shapiro–Wilk test), posteriorly One-Way ANOVA was used to compare the three groups, then comparison between pair were performed with Bonferroni test. * Significant difference with *p* < 0.05 respect to NP group.

It is well known that TIMP-1 regulates the metalloproteinases function in the heart, and in our study was shown that TIMP-1 was upregulated at both transcriptional and protein levels ( Figure 2; Figure 3) during LP and reversed in PP (7 days). 

In addition, TIMP-4 expression was significant greater in LP and PP compared with NP group (Figure 2). This supports that TIMP-1 and TIMP-4 upregulation in the heart during the pregnancy is associated with decreases in MMPs expression.

## 4. Discussion

This cardiac hypertrophy developed in the pregnancy is part of the cardiac remodeling reported by previous studies in rat and mice in similar physiological condition [13,14,15,16]. 

Our histological analysis in cardiac left ventricle of pregnant rats revealed the presence of various types of fibrosis, perivascular, pericardial, and interstitial; these changes in fibrosis content were reversed in postpartum. Several authors who have studied cardiac remodeling during pregnancy in the mice show that there is no presence of interstitial fibrosis [6,17]; in contrast, in rats there is only one study that reports interstitial cardiac fibrosis in pregnancy [18], but the increase with respect to the non-pregnant group was not significant. In our study using the Sprague–Dawley rat strain if significant differences were observed in the development of fibrosis during late pregnancy.

The presence of perivascular and pericardial fibrosis in the heart of pregnant rats that is showed in the present study has not been reported before. The fibrosis observed in our experiments could be the result from a compensatory effect in response to the increase in blood volume present in the pregnancy to improve the diastolic function. In addition, this remodeling that is present only in pregnancy and absent in postpartum could be associated with an increase in the collagen type III expression [11] and with a decrease in cardiac stiffness [4]. In the pregnancy-induced cardiac remodeling, adaptive changes are triggered to improve the cardiac function as we showed in this work; in contrast, in the pressure overload induced cardiac remodeling other alterations are performed as the increase in fibrosis associated to collagen type I deposition that compromise the cardiac function [19].

It is well known that the metalloproteinases system has an important role maintaining the structural integrity of the extracellular matrix in the heart. Its participation in various pathological conditions has been reported as well [20]. However, few studies exist that are oriented to understand its involvement in functional remodeling of the heart in non-pathological conditions as pregnancy. In the present study the MMP-1, MMP-2, and MMP-9 expression was lower in left ventricle of pregnant rats than in the non-pregnant group, while in the postpartum the MMP-1 and MMP-9 expression was similar to non-pregnant group; MMP-2 expression was less in postpartum group compared with non-pregnant group. The downregulation of metalloproteinases suggest its participation in the cardiac remodeling of the extracellular matrix during the pregnancy. In previous studies carried out in mice, MMP-3 upregulation in late pregnancy and immediate postpartum [21] and downregulation in the MMP-2, ADAM-15 (A disintegrin and metalloproteinase 15) and ADAM-17 (A disintegrin and metalloproteinase 17) transcript levels in late pregnancy were reported [6].

On the other hand, it is well known that TIMP-1 regulates the metalloproteinases function in the heart. In our study it was shown that TIMP-1 was upregulated at both transcriptional and protein levels during late pregnancy and reversed in medium postpartum (7 days). In addition, TIMP-4 expression was higher in late pregnancy and postpartum respect to non-pregnancy group. A recent study has reported that TIMP-1 is upregulated in the immediate postpartum (12 h after parturition) [20] while here we show that TIMP-1 expression is reversed after seven days postpartum. In general, it is known that TIMPs inhibit MMPs activity with different specificity, and TIMP-1 interacts with MMP-1 and MMP-2, while TIMP-4 interacts with MMP-9 [22]. In our study, TIMP-1 and TIMP-4 upregulation and MMP-1, MMP-2, and MMP-9 decreased in the heart during pregnancy, showing that the metalloproteinases system plays a key role in the extracellular matrix remodeling during pregnancy in the rat heart.

## 5. Conclusions

The present study showed the presence of perivascular, pericardial, and interstitial fibrosis in left ventricle of the rat heart, which is associated to downregulation of MMP-1, MMP-2, and MMP-9 and upregulation of TIMP-1 and TIMP-4 in late pregnancy. The changes of MMP-1, MMP-9, and TIMP-1 were reversed in postpartum of seven days. In contrast, decreased MMP-2 expression is associated with increased TIMP-4 expression during the postpartum. These data supported the participation of the metalloproteinase’s system in the regulation of synthesis and breakdown of the extracellular matrix components and improves the knowledge of the pregnancy-induced cardiac remodeling in an animal model.
